# Top-line Results from ESCAPE-LTE: An Open-Label Extension Study to Assess Long-term Safety of Esketamine Nasal Spray in Treatment Resistant Depression

**DOI:** 10.1192/j.eurpsy.2025.857

**Published:** 2025-08-26

**Authors:** Y. Godinov, D. J. Fu, C. von Holt, J. Buyze, R. Mehta, A. Reif, T. Messer, I. Bitter, R. Frey, L. Häggström, A. E. Anıl Yağcıoğlu, R. Nielsen, W. J. Cubała

**Affiliations:** 1 Janssen EMEA, Sofia, Bulgaria; 2 Janssen Research & Development LLC, New Jersey, United States; 3 Janssen EMEA, Neuss, Germany; 4 Janssen Pharmaceutica NV, Beerse, Belgium; 5 Janssen Inc., Toronto, Canada; 6 Department of Psychiatry, Psychosomatic Medicine and Psychotherapy, Goethe University Frankfurt, University Hospital, Frankfurt; 7 Fraunhofer Institute for Translational Medicine and Pharmacology ITMP, Theodor-Stern-Kai 7, 60596, Frankfurt am Main; 8 Danuvius Klinik GmbH, Technische Universität München, Pfaffenhofen an der Ilm, Germany; 9 Department of Psychiatry and Psychotherapy, Semmelweis University, Budapest, Hungary; 10 Department of Psychiatry and Psychotherapy, Medical University of Vienna, Vienna, Austria; 11 Affecta Psychiatric Clinic, Halmstad, Sweden; 12 Department of Psychiatry, Hacettepe University Faculty of Medicine, Ankara, Türkiye; 13 Aalborg University Hospital, Aalborg, Denmark; 14 Department of Psychiatry, Faculty of Medicine, Medical University of Gdańsk, Gdańsk, Poland

## Abstract

**Introduction:**

ESCAPE-LTE was a phase 4, long-term, open-label extension (OLE) study for patients (pts) with treatment resistant depression (TRD) who were continuing esketamine nasal spray (ESK-NS) treatment following ESCAPE-TRD. ESCAPE-TRD was a randomised, phase IIIb trial comparing the efficacy and safety of ESK-NS versus quetiapine extended release (Q-XR), when both were flexibly dosed alongside an ongoing selective serotonin/serotonin-norepinephrine reuptake inhibitor (SSRI/SNRI), in pts with TRD. The study demonstrated that ESK‑NS significantly increased the probability of achieving remission at Week 8, and of being relapse‑free through Week 32 after remission at Week 8, versus Q-XR (Reif *et al.* NEJM 2023; 389 1298–309).

**Objectives:**

To assess the long-term safety, tolerability and efficacy of ESK-NS alongside an SSRI/SNRI in pts with TRD. Here, the study design of ESCAPE-LTE is described; top-line results will be reported in the poster.

**Methods:**

ESCAPE-LTE was a single-arm, 2-year OLE to ESCAPE-TRD in 14 countries. Pts eligible for ESCAPE-LTE were those who completed ESK-NS treatment in combination with an SSRI/SNRI in ESCAPE-TRD through to the end of the study (Week 32), continued to benefit from the ESK-NS treatment regimen and for whom commercial ESK-NS was not accessible in their country. The starting dose of ESK-NS was based on the pts’ dose (28 mg [≥65 years and adults of Japanese ancestry], 56 mg or 84 mg) and frequency (weekly or biweekly) at completion of the maintenance phase (Week 32) of ESCAPE-TRD. Investigators were able to change the dose and frequency within label recommendations during the OLE based on clinical judgment.

The primary objective was to assess the long-term safety and tolerability of ESK-NS. The secondary objective was to assess the long-term efficacy of ESK-NS based on the proportion of pts being relapse-free at Week 104 (or end-of-study).

The primary endpoints were the number of pts who developed treatment-emergent adverse events, and suicidal ideation and behaviour, assessed by the Columbia-Suicide Severity Rating Scale (C-SSRS). Safety assessments also included body weight and vital sign measurements and clinical laboratory tests. The secondary endpoint was no relapse until the end of the prospective observation at Week 104 (or end-of-study); relapse was defined as a worsening of depressive symptoms as indicated by a total Montgomery-Åsberg Depression Rating Scale (MADRS) score of ≥22 at two consecutive assessments; hospitalisation for worsening depression, suicide prevention or attempt; or any other event assessed by the investigator to be indicative of relapse.

**Results:**

183 pts were enrolled in the ESCAPE-LTE. Top-line study results will be reported in the poster.

**Conclusions:**

Results from the ESCAPE-LTE will provide evidence for the long-term safety, tolerability and efficacy of ESK-NS as a treatment for pts with TRD.

**Disclosure of Interest:**

None Declared

